# Bread consumption trends in Poland: A socio‐economic perspective and factors affecting current intake

**DOI:** 10.1002/fsn3.4383

**Published:** 2024-08-11

**Authors:** Adam Sadowski, Bogusława Dobrowolska, Piotr Dziugan, Ilona Motyl, Wiktoria Liszkowska, Izabela Rydlewska‐Liszkowska, Joanna Berłowska

**Affiliations:** ^1^ Department of Strategy and Value‐Based Management University of Lodz Lodz Poland; ^2^ Department of Environmental Biotechnology, Faculty of Biotechnology and Food Sciences Lodz University of Technology Lodz Poland; ^3^ Department of Medical Insurance and Health Care Financing, Faculty of Health Sciences Medical University of Lodz Lodz Poland

**Keywords:** bread consumption, consumer acceptance, consumption trends, dietary patterns, nutrition

## Abstract

Fermentation processes have been known since ancient times and are widely used in the production of food, beverages, and other areas. One of the most well‐known fermented products is bread. It plays an important role in human nutrition because of the valuable compounds it contains. The growing population leads to an increase in global bread consumption and other bakery products. Simultaneously, in developed countries, declining consumption trends are observed. Understanding the complex interplay between socio‐economic dynamics, food production policies, and the dietary patterns of society is crucial for shaping effective strategies that align with the principles of a sustainable and resilient food system. The aim of this study was to determine bread consumption trends in Poland. Data were obtained from the Household Budget Surveys carried out by Statistics Poland. The study considered the period from 1978 to 2020 and was performed in three dimensions: socio‐economic groups, the number of people in households, and income quintile groups. A decreasing trend in bread consumption was observed within the analyzed period in all three socio‐economic groups. Based on the analysis of the relationship between bread consumption and income level per capita, the same observation was made. There was no correlation between the amount of bread consumed and the number of people in the household. The obtained data are determined by many factors, including prices of bread, demographic changes, dietary patterns, and education in the field of human nutrition.

## Graphical abstract

Understanding the complex interplay between socio‐economic dynamics, food production policies, and waste management is crucial for shaping effective strategies that align with the principles of a sustainable and resilient food system. It is also important to correlate consumption with current trends, nutritional education, prices, and demographic changes. According to the multidimensional analysis of the current intake of bread in Poland, a decreasing trend in consumption was observed in Polish society.

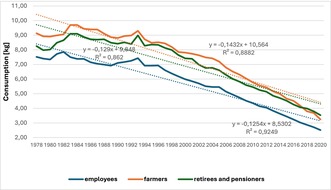

## 1 INTRODUCTION

Bread, a staple in diets worldwide, has been a fundamental food item throughout human history (Ibrahim et al., 2015). It is a significant high‐calorie source of nutrients such as proteins, B vitamins, and minerals (Chlopicka et al., 2012). The consumption of bread varies significantly across regions, influenced mainly by the availability and cost of grain. This variation is visible in European countries, with the UK and Turkey exemplifying contrasting consumption patterns: the UK's annual per capita bread consumption is 37 kg, whereas in Turkey, per capita consumption stands at 150 kg (Quilez & Salas‐Salvado, 2012; Sarica et al., 2021). In Africa and Asia, where it was not traditionally a staple, consumption of bread has increased dramatically, driven by the time‐savings and convenience it offers (Weegels, 2019). In contrast, there has been a significant decrease in the consumption of bread per capita in Europe (Fabijańska & Fronczyk, 2015; Hadyński & Genstwa, 2019). Although bread is still considered one of the most representative food and beverage products consumed in the EU‐27, its consumption in the basket of products amounts to 19.75 Mt per year, which translates to 39.3 kg per capita annually, accounting for only 7.3% of the overall basket (Notarnicola et al., 2017). Bread is also one of the most wasted types of food, not only at the consumption stage but also at other points along the value chain, including bakery product producers and distributors (Kowalewska & Kołłajtis‐Dołowy, 2018; Pietrangeli et al., 2023; Weber et al., 2023).

The 21st century is bringing many changes, especially in the hierarchy of values and the establishment of priorities. The rapid progress in globalization and improving access to information have led to more conscious consumers, as tradition is being replaced by information (Sorin et al., 2018). This is reflected in basic decision‐making, such as choosing food products. Polish consumers, for instance, following a similar trend to Germans, are increasingly scrutinizing commercial information about bread, which is directly impacting their purchasing decisions. This has been underscored by a labelled choice experiment with different breads conducted in Germany. Three types of bread—genetically modified (GM), conventional, and organic—were considered in the context of consumer choice. The findings highlighted that consumers who accepted GM bread were younger, less educated, and did not pay attention to nutritional habits. In general, GM bread was viewed as unhealthy, and consumers were unwilling to pay high prices compared to organic or conventional bread (Wuepper et al., 2019).

The aim of this research was to determine trends in bread consumption and their relation to socio‐economic groups, the number of people in households, and income quantile groups. The time periods for the analyses in these three dimensions were dictated by the availability of the official statistical data from the Household Budget Surveys carried out by Statistics Poland. This is the first comprehensive study showing trends in bread consumption in Poland.

### 1.1 Theory

Bread became an integral part of the daily diet and had cultural significance, often playing a role in religious rituals. The first mention of bread fermentation derives from the Neolithic period. The attempts at fermentation may had been accidental when people noticed changes in food ingredients left for a longer time. Later, in ancient times, the fermentation of flour and water became the precursor of a process that influenced the shelf‐life, taste, and nutritional value of bread. Its development took place initially in the Fertile Cresent region in the Middle East and a little later in the Mediterranean, especially in ancient Greece and Rome. Long before animals were raised and farmed, people gathered and ate cereal grains such as wheat and barley. The use of sourdough in bread production not only influenced the durability of this food product in the Fertile Cresent climatic conditions but also gave it a characteristic taste. In Mesopotamia, considered one of the centres of civilization development, sourdough was used in the baking process of bread over 4000 years ago. Archaeological discoveries indicate the presence of bread ovens and various types of bread, which proves the developed practice of baking (William, 2011). In Polish culture, bread is not only a food product but also a tradition. Bread has traditionally been treated with respect and even reverence, serving as a symbol of prosperity. Bread also plays an important role in the Christian religion, symbolizing the connection between God and people (Fabijańska & Fronczyk, 2015; Kowalska, 2015). Therefore, there is a strong tradition of baking and bread production.

The global consumption of bread and other bakery products is continuing to increase. This is primarily due to the growing population. However, at the same time, there is a decrease in the amount of bread consumed per capita in developed countries (including the total level of flour used as a raw material in baking) (AIBI, 2015; GOV.UK, 2019; Shahbandeh, 2021, 2022). This trend is also visible among consumers in Poland. There are many scientific studies describing the factors affecting changes in demand for bread in Poland. One criterion is the demand for high‐quality food (Żakowska‐Biemans & Gutkowska, 2018). Access to reliable information, the latest dietary trends promoted by health and nutrition organizations such as the World Health Organization (WHO) and the Polish National Food and Nutrition Institute, as well as extensive marketing campaigns led by dietitians and companies providing dietary catering services, are raising awareness about healthy dietary habits and preventative health (Gil et al., 2022). Additionally, the significant increase in the number of large supermarkets offering a wide range of food products has led to the introduction not only of high‐quality products but also of many products of questionable quality and nutritional value (Hadyński & Genstwa, 2019; Kucharska, 2016). For example, the bread sold in supermarkets differs significantly in terms of quality and nutritional properties from traditional bread (Jeżewska‐Zychowicz & Królak, 2015). For this reason, consumers have started to read product labels more closely to avoid processed products, paying attention to the content of salt, simple sugars, and fats, as well as food additives such as preservatives, leavening agents, emulsifiers, and artificial dyes (Develey, n.d.; Jeżewska‐Zychowicz & Królak, 2020; Narodowe Centrum Edukacji Żywieniowej, n.d.).

Another very important factor closely related to the consumption of quality food products, including bread, is income. Thilmany and coworkers showed a correlation between dietary trends and social classes. They observed that consumer behaviours regarding food strongly depend on budget. Individuals with lower incomes tend to choose products at affordable prices (which often have lower quality). In contrast, more affluent individuals buy more expensive products, often produced by local suppliers, which are characterized by significantly higher quality (Thilmany et al., 2008). Moreover, it is proved that individuals with higher social status seek not only to satisfy their hunger but also new culinary experiences, often replacing products such as wheat bread in their daily diets (Babicz‐Zielińska & Jeżewska‐Zychowicz, 2015; Żelazna et al., 2002). Frequent dining at restaurants also contributes to changing dietary habits. On the other hand, less affluent consumers view bread as a staple product that satisfies basic nutritional needs.

Apart from the nutritional aspects of food, an essential condition for conscious economic activity is an understanding of the principles governing economic relations, both from the perspective of the national economy as a whole and within individual businesses. Knowledge of economic principles is crucial for developing marketing and product strategies. Understanding economics is also important for innovation management. By learning about the factors creating demand for a particular product, a company can engage in innovative processes that meet anticipated consumer needs and behaviors (Jasiński, 2020). This also applies to the bakery industry. It is already possible to find bread producers who, guided by consumption trends, are directing their production toward currently niche areas with a growing market share (Godula et al., 2019; Jurczuk et al., 2018).

### 1.2 Methodology

Bread consumption was analyzed based on data from Household Budget Surveys, carried out by Statistics Poland. The analysis was performed across three dimensions: socio‐economic group, number of individuals in the household, and income quintile group. The time frames for these analyses were dictated by the availability of Statistics Poland data. The data span the years 1978–2020. Both cross‐sectional and time‐series samples were utilized. The study population was divided into three socio‐economic groups: workers, farmers, and retirees and pensioners:

- Employee households: households whose exclusive or primary (predominant) source of income comes from work in the public or private sector, including physical labour and contract work. Additional sources of income for these households may include pensions, disability benefits or other non‐earnings sources, self‐employment (excluding individual agriculture), practicing a profession, or cultivating agricultural land up to 1.00 ha in size. Income from additional sources is lower than income obtained from salaried work.
- Farmer households: households whose exclusive or primary source of income comes from a farm with agricultural land exceeding 1.00 ha (including users of plots of up to 1.00 ha of agricultural land and owners of livestock without agricultural land if these are the exclusive or primary source of income). Additional sources of income for these households may include pensions, disability benefits, or other non‐earnings sources; self‐employment outside agriculture; or practicing a profession. Income obtained from additional sources is lower than income obtained from farming.
- Retiree and pensioner households: households whose exclusive or primary source of income comes from a pension or disability benefit. Additional sources of income may include other non‐earnings sources, apart from the pension or disability benefit, such as income from property, assistance, gifts, salaried work, use of agricultural land or plots, self‐employment outside of agriculture, or practicing a profession. Income obtained from additional sources is lower than income obtained from pensions and disability benefits.

Households were divided based on the number of residents (one, two, three, four, five, six, or more), over the years 1995–2020. The households were further divided into quintile groups, which were delineated based on disposable income (expenditure) per person in the household, as an indicator of household affluence. For example, individuals belonging to the first quintile group comprised the bottom 20% in terms of income and were considered to belong to the least affluent households. The analysis of data according to income covered the years 2006–2020. Having access to long‐time series allowed for the assessment of changes in bread consumption over time. For this purpose, trend function analysis was utilized, along with an assessment of level and diversity, which was performed using box plots.

## 2 RESULTS

**FIGURE 1:**
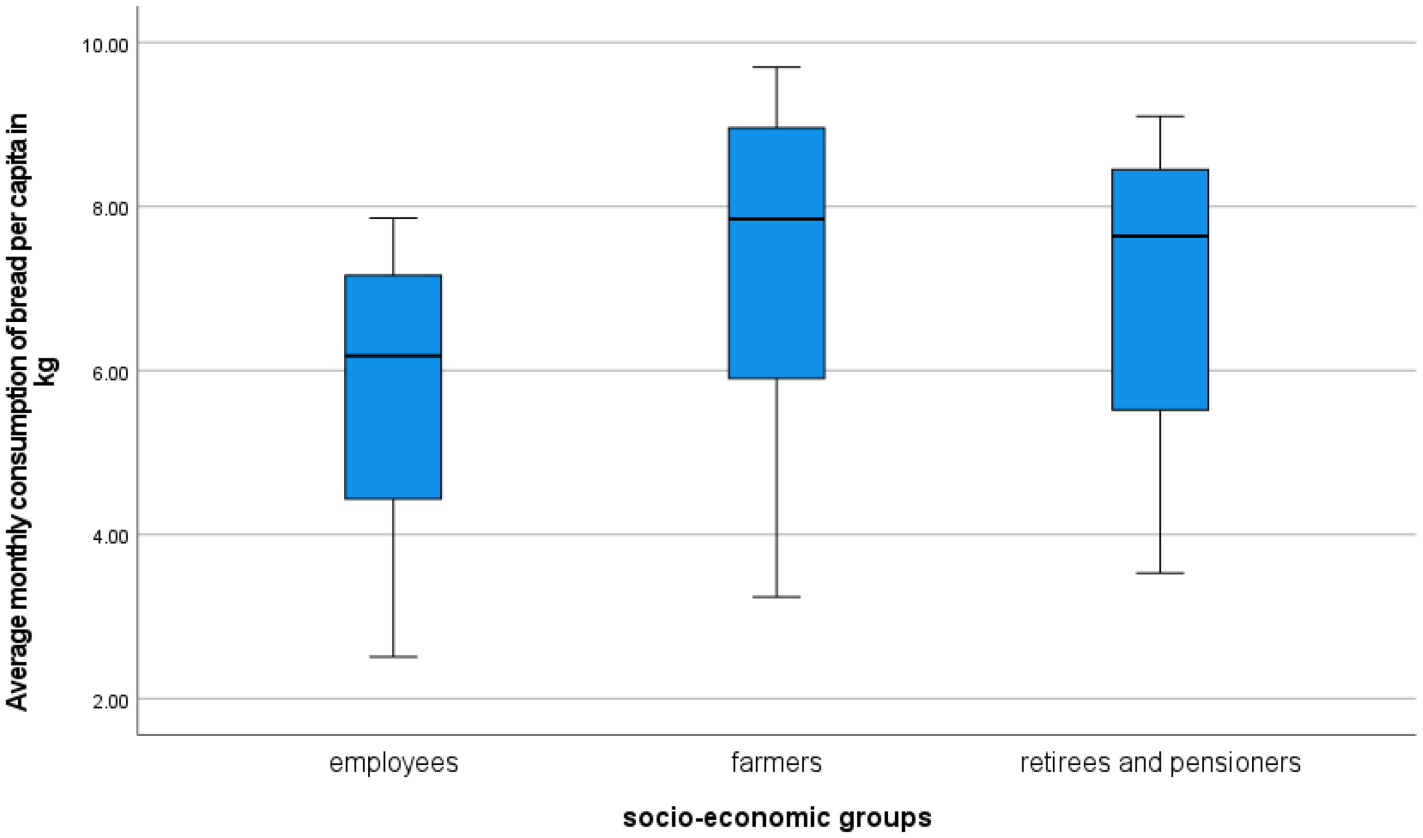
Box plots of average monthly bread consumption per person in kilograms by socio‐economic groups of households in Poland from 1978 to 2020. Source: Own preparation based on Household Budget Survey (Główny Urząd Statystyczny, n.d.).

**FIGURE 2:**
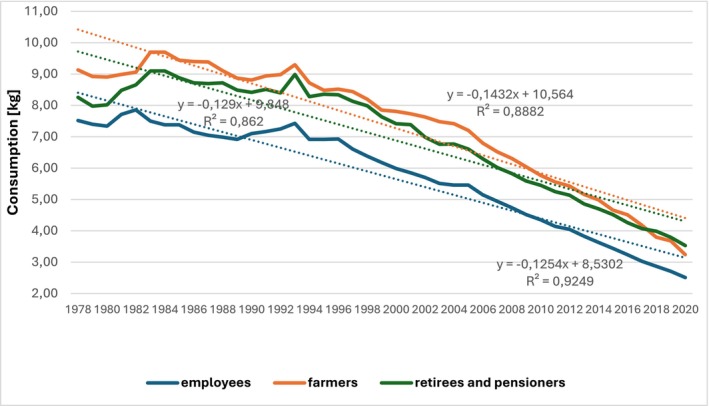
Average monthly bread consumption per person in kilograms by socio‐economic group in Poland from 1978 to 2020. Source: Own work based on the Household Budget Survey (Główny Urząd Statystyczny, n.d.).

The differences in consumption become apparent when analyzing individual types of households. Belonging to a specific type of household determines the quantity of bread consumed. A comparison of employee households, agricultural households, and retiree and pensioner households reveals clear disparities in consumption levels. Figures 1 and 2 indicate the variation in bread consumption in Polish households based on socio‐economic group. Predictably, agricultural households, as well as retirees and pensioners, were found to consume the most bread, while employee households consumed the least.

The average monthly bread consumption per person in Poland is decreasing, as indicated by the linear trend coefficients presented in Figure 2. From 1978 to 2020, the average monthly bread consumption per person decreased by an annual average of 0.14 kg in agricultural households, and by 0.13 kg in retiree and pensioner households as well as employee households. The data from 2020 confirm research conducted on a group of 112 women and men, which showed that during the COVID‐19 pandemic lockdown, bread consumption decreased, especially among men. However, white bread remained one of the main components of the diet (Bolesławska et al., 2021; Xiong et al., 2021). Simultaneously with the decline in bread consumption during the COVID‐19 pandemic, there was a significant decrease in the amount of bread waste generated by households (Çavuşa et al., 2022).

**FIGURE 3:**
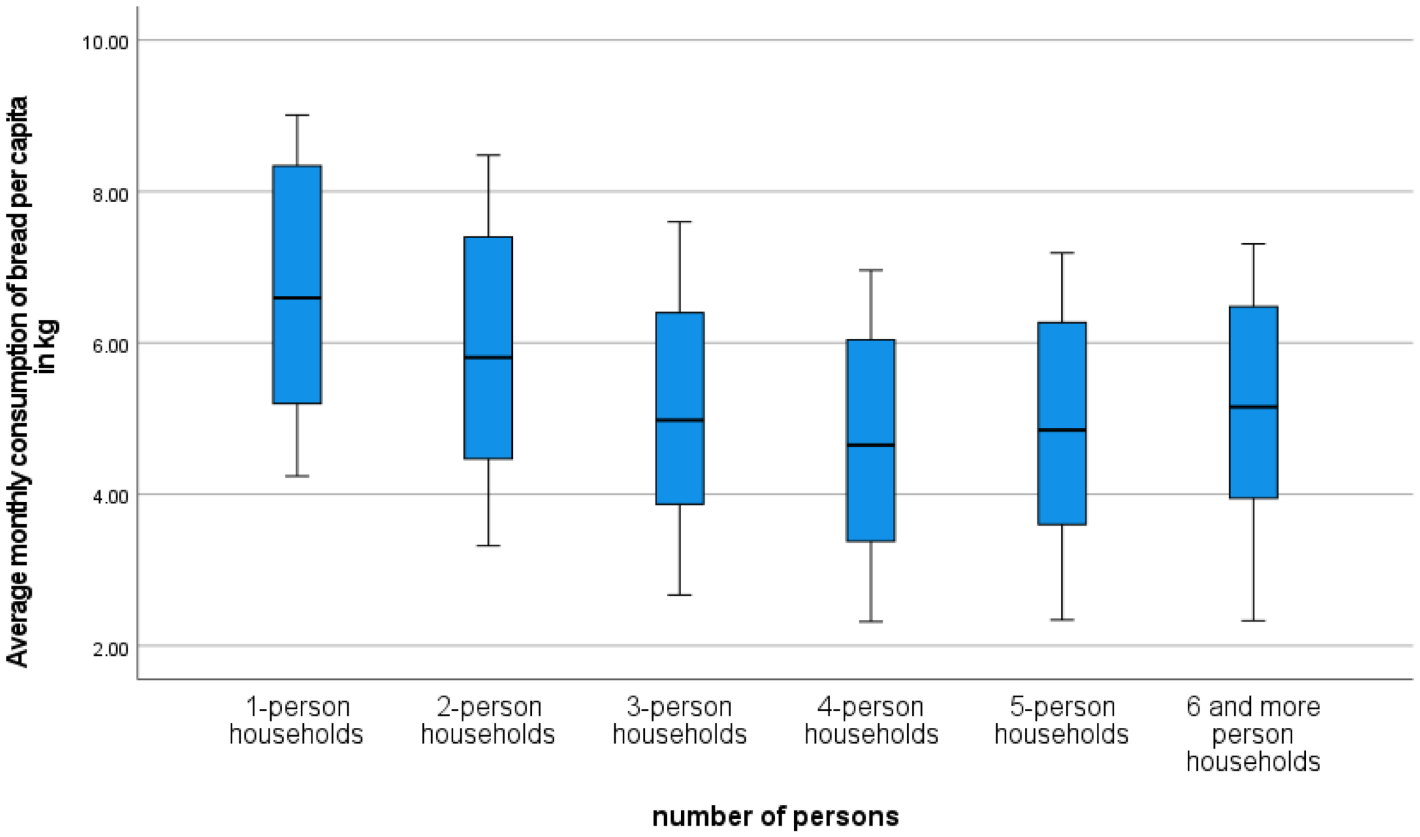
Box plots of average monthly bread consumption per person in kilograms by household size groups in Poland from 1995 to 2020. Source: Own work based on Household Budget Surveys (Główny Urząd Statystyczny, n.d.).

**FIGURE 4:**
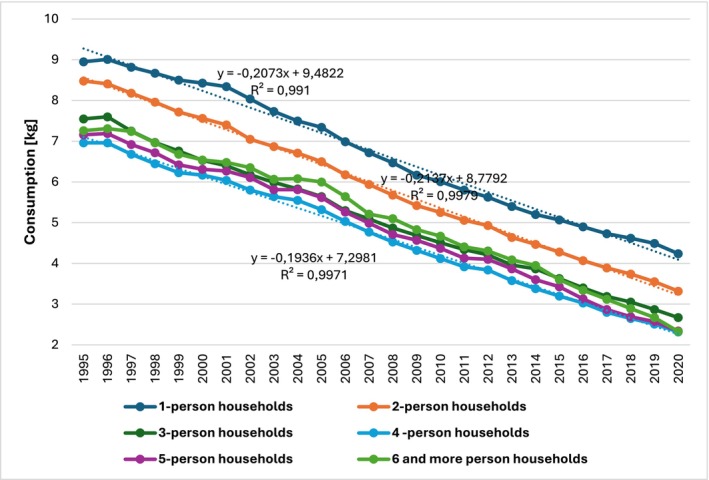
Average monthly bread consumption per person in kilograms by the number of people in households in Poland from 1995 to 2020. Source: Own work based on Household Budget Surveys (Główny Urząd Statystyczny, n.d.).

An interesting conclusion can be drawn from the analysis of bread consumption based on household size presented in Figures 3 and 4. Given the available statistical data, the analysis covered the years 1995–2020. Single‐person households consumed the most bread, while four‐person households consumed the least. The “scale effect” does not seem to apply here. The analysis of trend function coefficients presented in Figure 4 indicates that from 1995 to 2020, the average monthly bread consumption per person decreased annually from 19 dag for four‐person households to 21 dag for one‐ and two‐person households.

**FIGURE 5:**
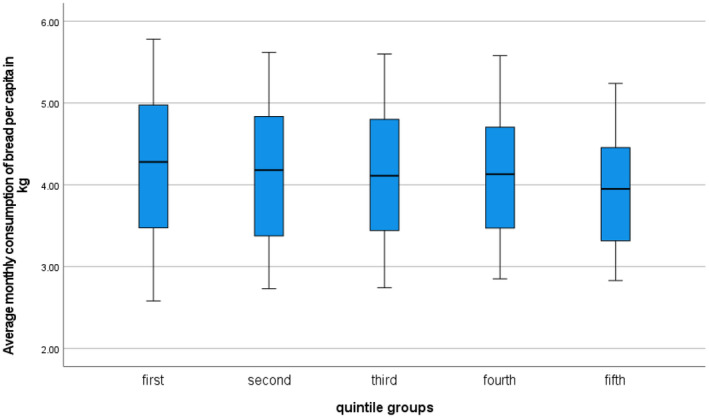
Box plots of average monthly bread consumption per person in kilograms according to quintile income groups of households in Poland from 2006 to 2020. Source: Own work based on Household Budget Surveys (Główny Urząd Statystyczny, n.d.).

**FIGURE 6:**
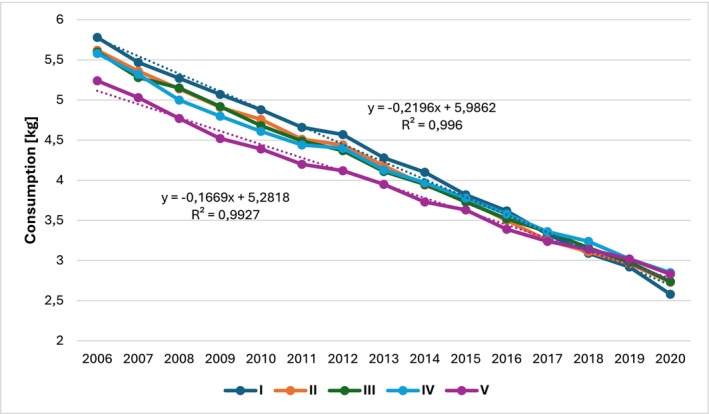
Average monthly bread consumption per person in kilograms according to quintile income groups of households in Poland from 2006 to 2020. Own preparation based on Household Budget Surveys (Główny Urząd Statystyczny, n.d.).

The analysis of bread consumption in Poland based on household affluence was conducted using data from Statistics Poland published in Household Budget Surveys for the years 2006–2020. The results were presented in Figures 5 and 6.

Overall, bread consumption decreased as household affluence increased. The average bread consumption per person in households in the first quintile group (the poorest) was on average 4.23 kg, while in households in the fifth quintile group (the wealthiest), the average was 3.95 kg. This pattern indicates that bread is primarily used by less affluent residents as a main source of energy. The trend function coefficients shown in Figures 5 and 6 indicate that from 2006 to 2020, the average monthly bread consumption per person decreased annually from 22 dag in the poorest households to 17 dag in the wealthiest households. When considering the correlation between bread consumption and the primary source of energy, studies show that a similar relationship exists between less and more developed countries (Truesdell et al., 2018).

## 3 DISCUSSION

Bread is not only considered as a staple food. It is important to note that the consumption of bakery products also provides valuable nutrients such as carbohydrates (e.g., starch), fibre, protein and vitamins and minerals (such as calcium or iron) (Lockyer & Spiro, 2020). In Europe, the average consumption of bread and bakery products per capita is approximately 57 kg annually. Despite significant variations in bread consumption patterns across the European Union, in most countries, the average consumption of bread per person is around 50 kg annually. Countries such as Germany and Austria have the highest annual bread consumption per capita, at around 80 kg, while the United Kingdom and Ireland consume less than 50 kg annually per person. Spain and France also have high consumption rates. Although in some European Union countries, such as Poland, bread consumption is declining, it is anticipated that the market will continue to grow during the next decade (European Comission, 2021).

In less developed countries, the intake of bakery products is higher. Among Polish people, the bread consumption tends to decrease, as it is in developed countries. This study indicates that the type of socioeconomic group may have an influence on the consumption of bakery products. This may be reflected in the disparate levels of education among these groups. Furthermore, introducing healthier eating habits and increasing awareness of caring for the environment have an impact on the amount of bread purchased and consumed. Consumers tend to favour the authenticity of food, tradition, the natural surroundings, the standard of living, and overall well‐being. Considering all these elements, the food sector in Poland has met the needs of its customers. The biggest change in the manufacturing process is the introduction on the market of innovative products enriched with diverse nutrients, especially dietary fibre (Królak et al., 2022). However, in addition to obtaining high‐value products, the method of processing and the selection of technology play an important role. Consumers express their concerns about the use of new technologies in food production, which include threats to the environment, health, naturalness of food and quality of life (Sajdakowska et al., 2018). Therefore, ecological processes are increasingly popular in society. In conjunction with the escalating rationalization, this may lead to a greater social awareness of the environment, which is important in the context of the production of waste, both in the industrial sector and in households. There are at least a few factors affecting bread consumption worldwide which may have an impact on bread consumption, and which should affect the scope of further research in the field of bread consumption.

### 3.1 Prices

The recent Harmonized Index of Consumer Prices by Eurostat revealed a significant increase in bread prices. This has been primarily due to the Russia‐Ukraine conflict, which has significantly disturbed global markets, as Russia and Ukraine are both significant exporters of grains, wheat, corn, oilseeds (especially sunflower seeds), and fertilizers. In August 2022, the average price of bread in the European Union was 18% higher than in August 2021. This marks a substantial increase compared to August 2021, when the price of bread was on average 3% higher than in August 2020. Core inflation has also increased significantly, from +3% to +10% (Eurostat, 2022). The rising prices may partially limit the consumption of some food products. For example, an increasing number of households in Poland are making bread at home to save money. This also has the effect of decreasing sales indicators for bread in Poland (Ekonomia, n.d.).

Within the analyzed groups based on income level, there is a tendency to show that the poorer people consume the most bread. It is also noticeable that even if prices increase, the poorest people consume more bread. This interesting trend has been described in other parts of the world. Studies conducted by Engindeniz and Bolatova on a group of 269 families from Kazakhstan and Turkey revealed that wealthier families consumed smaller quantities of bread (Engindeniz & Bolatova, 2019). Considering consumption within defined socio‐economic groups, this trend is also confirmed. In this study, it was shown that farmer households and retirees and pensioners households presented increased bread consumption compared to the employees group. This can be related to the lower incomes in these two groups. According to the Social Insurance Institution and Agricultural Social Insurance Fund, in 2006 in Poland, the minimal pension was equal to 597.46 PLN, and in 2020, it was equal to 1200 PLN (Kasa Rolniczego Ubezpieczenia Społecznego, n.d.; Składki ZUS n.d.; Wolters Kluwer, 2006). However, the average monthly pension published by The Social Insurance Institution and Agricultural Social Insurance Fund differed and stood at 1523.65 PLN in 2006 and 2564.54 PLN in 2020 for pensioners and 850.47 PLN in 2006 and 1383.58 PLN for farmers (Departament Statystyki I Prognoz Aktuarialnych, 2020; Główny Urząd Statystyczny, 2021a; Zakład Ubezpieczeń Społecznych, 2007; Zakład Ubezpieczeń Społecznych DS i PA, n.d.). These values are significantly lower than the minimum monthly salaries in the indicated years, which were at the level of 899.10 PLN in 2006 (average monthly salary of 2477.23 PLN) and 2600 PLN in 2020 (monthly average of 5167.47 PLN), so it could suggest that less affluent individuals would consume more food products considered to be cheaper (Electronic ZUS, 2023; Elektroniczny ZUS, 2023). However, in this study, such a trend has not been observed within the analysis of bread consumption.

### 3.2 Environmental management

Another factor reducing the consumption of certain food products, including bread, is increasing awareness related to environmental management. This leads to a rationalization of consumption, with the goal of limiting the generation of food waste. Food waste is a global problem with various negative consequences. As early as 2011, the Food and Agriculture Organization of the United Nations estimated that one‐third of all food produced is lost or wasted (Food and Agriculture Organization of the United Nations, 2011). This can be a challenge from an environmental and socio‐economic perspective, particularly in terms of costs not only to the producer but also to the final customer. The issue of managing food waste has developed on an unprecedented scale, much faster than the development of disposal technologies. The connection between food waste, technological resources (especially water and energy), environmental impact, and social justice highlights the need for effective and rational waste management (Kibler et al., 2018; Zabłocka et al., 2016). Data published in 2021 (and updated in 2023) by Eurostat reveals that the European Union generates approximately 59 million tons of food waste, with a market value of 132 billion euros. It has been calculated that 131 kg of waste per capita is produced annually (Eurostat, 2023). This issue is even more serious given that, according to the same data, in 2022, approximately 33 million people in the European Union were living on incomes sufficient to afford a healthy, well‐balanced meal only every other day (Eurostat, 2022b). More detailed research results concerning household‐generated bread waste are limited and difficult to generalize, due to methodological differences. In Norway, 0.44 kg of fresh bread is wasted per household per week (Hanssen et al., 2016). In Italy, this figure stands at 68 g per family per week (Scalvedi & Rossi, 2021). In the Netherlands, 9.2 kg is wasted per person annually (van Dooren et al., 2019). Furthermore, studies conducted in Australia showed that the number of people living in a household is positively correlated with the volume of waste generated per capita (Ananda et al., 2022).

Research on socio‐economic factors related to environmental awareness is limited and inconsistent. In the case of Poland, there is no statistically significant difference between individuals with lower/basic and higher education and their consumption behaviours (Boylan et al., 2011). However, studies conducted in other countries revealed a strong correlation between education and income level and environmental caring patterns. Individuals with higher education tend to comprehend environmental issues better and thus become more concerned about environmental quality and engaged in environmentally responsible activities (Strieder Philippssen et al., 2017; Thompson et al., 2020).

### 3.3 Demographic shift

A significant global demographic shift is currently underway, characterized by an increase in single‐person households, which are also aging. There is a rise in mobile and flexible employees who embrace modern lifestyles that foster an appreciation for foreign cultures and culinary diversity (Eglite & Kunkulberga, 2017). In Poland, belonging to the given socio‐economic group strongly determines dietary habits. Currently, almost 60% of people are of working age (18–59/64 years old), and the average age is around 40 years old (Lisiak & Morytz‐Balska, 2023). Although in this study, bread consumption was the lowest in the group of employees, their dietary habits can influence their intake of food products. Stosovic and coworkers investigated the dietary habits of Serbian older adults (≥65 years) by comparing them with younger adults (18–64 years). They found that older people tend to eat healthier and have better dietary habits, such as eating a regular breakfast or choosing brown and whole‐grain bread (Stosovic et al., 2021). Hanus demonstrated that the phenomenon of rationalization is evident in Poles eating habits and is associated with a higher awareness of food products. The amount of food consumed is affected by the lower consumption of most food articles, the higher importance of quality and consistency of food, the development of the phenomenon of food sharing, and the growing popularity of vegetarians or vegans (Hanus, 2021). This shift is further compounded by a lack of dynamic growth in developed countries, which results in stagnant household incomes and a lower demand for bread and bakery products. In their study, Szczepańska and coworkers showed that wholemeal bread was eaten more often than white bread, 20.7% and 27.65%, respectively. Suliga presented a diverse set of findings, assessing the health‐related eating behaviours of both adult and elderly individuals. They reported that 17.7% of the 166 respondents consumed wholemeal bread several times daily (16.6% of women and 19.4% of men), and 15.9% of respondents (14.4% of women and 17.9% of men) reported consumption of this product several times a week (Szczepańska et al., 2020). It can be associated with growing consumer preferences, especially for products with value added (snacks, convenience food) and food consumed outside the home. This prompts producers to enhance bakery products by incorporating beneficial ingredients such as seeds or dietary fibre. The natural form of fibre (sunflower seeds) proved to be more convincing to consumers than a micronized fiber enrichment, even if the grains used contained less fibre. Jeżewska‐Zychowicz and coworkers found that only one‐third of the study sample said they would eat rolls with pure fibre added. Furthermore, those who emphasized the importance of fibre, grains, or wholemeal flour addition when choosing bread were more likely to indicate their intention to consume the roll topped with sunflower seeds. The limited information on fibre application on food labels influenced the selection of rolls for consumption. However, the ability to identify the source of dietary fibre proved to be more significant when individuals declared their intention to consume a roll. Hence, these findings indicate the necessity of establishing educational initiatives aimed at enhancing knowledge, leading to the selection of high‐fibre foods. Furthermore, the importance of fibre content during the selection of cereal products highlights the importance of such factors. The results regarding the roll enriched with fibre show that information about fibre content on the label is going to increase consumers' awareness about bread (Jeżewska‐Zychowicz & Królak, 2020).

### 3.4 Education in the field of human nutrition and bread consumption

Education in the field of human nutrition plays a significant role in consumption trends. When combined with conscious practices aimed at rationalizing consumption and dietary habits, consumer awareness will gradually increase. The socio‐economic groups and the income per capita strongly influence nutrition. In this study, the biggest bread consumption was presented in the group of farmers. The same observation was made for the income level per capita. It was noticed that the poorest people consume more bread than wealthier people. This can be related not only to financial limitations but also to nutritional education. In the mass media, confusing messages concerning bread and its supposed negative effect on human health are spread. This is in contrast to the fact that bread contains a lot of valuable micro‐ and macroelements (Çobanoğlu et al., 2022; O'Connor, 2012).

Studies also show that consumers still don't know enough about where food products and food additives come from. A study conducted in the UK revealed that, despite its importance as a nutritious, versatile, inexpensive, and convenient carbohydrate, bread sales in the country have declined over the past 50 years. Popular beliefs about bread and health can have a significant impact on consumers' negative attitudes toward them. It is believed that bread and other starchy products can contribute to weight gain, and that consuming bread can cause bloating or other gastrointestinal symptoms in the general healthy population. Additionally, emerging trends in “natural” and organic food, such as simple and healthy food compositions transparently presented on labels or food made from “natural” ingredients, have a significant impact on the food sector. Recent trends indicate that food is increasingly being sourced from local producers. The utilization of environmentally sustainable products and the marketing of food based on dietary preferences also contribute to altering consumers' perception of the conventional loaf of bread as being “processed” (Lockyer & Spiro, 2020).

Many factors affect bread consumption. This is related to socioeconomic groups, rising bread prices, and growing awareness of human nutrition. Even though bread consumption in Poland is decreasing, it should be kept in mind that it is still a primary source of many valuable micro‐ and macroelements. Therefore, in the future, it is important to broaden knowledge about consumer behaviours and factors determining their food choices.

### 3.5 Summary

Recent changes in household consumption reflect the profound socio‐economic transformations occurring in highly developed countries. Consumption is now regarded by many economists as the most powerful productive force, which explains the growing interest in this sphere from both academia and business, including small and medium‐sized companies such as bakeries (Komor et al., 2020). Currently, white bread made from wheat flour is losing market share. The key determinants influencing the purchase of bread are related primarily to the characteristics of the product. Scientific studies indicate that consumer choices are affected not only by nutritional considerations but also by attributes such as taste, freshness, and price (Drugova et al., 2020). A previous study conducted in Sweden suggests that the most crucial feature influencing the purchase of organic bread is taste. Respondents also indicated that although organic bread is perceived as more expensive, it is considered healthier compared to white bread (Magnusson et al., 2001). As consumers become more aware of the health aspects of their diets, there is growing attention to the potential implications associated with consuming basic food products, such as white bread. Many studies on the impact of wheat bread consumption show a negative influence on health, including an increased tendency to obesity, an increase in body mass index (BMI), and a higher abdominal fat content (Romaguera et al., 2011). The study ‘Health, Alcohol, and Psychosocial Factors in Eastern Europe (HAPPIE)’ revealed a positive correlation between bread intake and cancer mortality in Poland (Stefler et al., 2021). In 2020, the average Polish consumer spent 50.48 PLN on grain products each month, with 18.65 PLN spent on bread (Główny Urząd Statystyczny, 2021b).

Despite the decline in bread consumption in Poland and other countries, it should be kept in mind that cereal products are a source of many beneficial micro‐ and macronutrients (Carocho et al., 2020; Torrinha et al., 2019). As an easily accessible food product, bread should remain in the daily diet. In addition, bakeries have begun to use various types of additives that increase the nutritional value of bread or provide a partial substitute for flour (Baker et al., 2022; Dziki et al., 2022; Kahlaoui et al., 2022; Mafu et al., 2022). Research by Żakowska‐Bielmans and Kostyra indicates that adding dietary fiber from oats, a healthy supplement, to bakery products does not impact the willingness of Polish consumers to purchase wheat‐rye bread. However, oat additives can enhance the bread's nutritional value (Żakowska‐Biemans & Kostyra, 2023). There are also studies showing the beneficial effects of certain types of bread made from different cereals. Olagunju and coworkers have proven that in vitro multigrain breads can have antioxidant and antidiabetic effects (Olagunju et al., 2021).

## 4 CONCLUSION

This research has provided the first comprehensive study of trends in bread consumption in Poland. The study considered the period from 1978 to 2020. The analysis was performed in three dimensions: socio‐economic groups, the number of people in households, and income quintile groups. A persistent decreasing trend in bread consumption was observed over time. This trend applied to all examined socio‐economic groups, and was most noticeable in the case of households of farmers and agricultural workers. Significant differences in the amounts of bread consumed were also observed depending on the type of household. Bread tended to serve as a primary source of energy for farmers and agricultural workers, whereas for other types of households, it was only a supplementary source of energy. Defining the specific role that bread plays depending on the type of household requires further in‐depth empirical research.

The analysis of bread consumption based on household size covered the years 1995–2020. Single‐person households consumed the most bread, while four‐person households consumed the least.

The income level did not significantly influence the amount of bread consumed. During times of economic growth, as incomes rise, there may be a decrease in the quantity of bread consumed. However, this does not necessarily mean that consumers spend less on bread, as they may purchase more expensive types. More work is needed to explore the relationship between expenditure on bread and incomes in different types of households. In future studies, it is also worth to analyse the consumption of ready‐made foods such as hamburgers or hotdogs, as they are based on cereal goods. Their consumption is determined by various factors, but their impact on bread consumption can be further examined.

## AUTHOR CONTRIBUTIONS

**Adam Sadowski:** Conceptualization (equal); formal analysis (equal); methodology (equal); supervision (lead); writing – review and editing (equal). **Bogusława Dobrowolska:** Conceptualization (equal); data curation (lead); formal analysis (equal); methodology (equal). **Piotr Dziugan:** Conceptualization (equal); investigation (equal); writing – original draft (equal). **Ilona Motyl:** Investigation (equal); writing – review and editing (equal). **Wiktoria Liszkowska:** Investigation (equal); writing – original draft (equal); writing – review and editing (equal). **Izabela Rydlewska‐Liszkowska:** Writing – review and editing (equal). **Joanna Berłowska:** Conceptualization (equal); project administration (lead).

## CONFLICT OF INTEREST STATEMENT

The authors declare no conflicts of interest.

## Data Availability

The data that support the findings of this study derive from Household Budget Surveys carried out by Statistics Poland and are available in the public domain: https://stat.gov.pl/en/.
